# Characterization and Evaluation of the Pathogenicity of a Natural Gene-Deleted Transmissible Gastroenteritis Virus in China

**DOI:** 10.1155/2023/2652850

**Published:** 2023-03-03

**Authors:** Lei Xu, Hong-bo Dai, Zhi-peng Luo, Ling Zhu, Jun Zhao, Feng-qin Lee, Ze-yan Liu, Min-cai Nie, Xue-tao Wang, Yuan-cheng Zhou, Zhi-wen Xu

**Affiliations:** ^1^College of Veterinary Medicine, Key Laboratory of Animal Diseases and Human Health of Sichuan Province, Sichuan Agricultural University, Chengdu, China; ^2^Veterinary Biologicals Engineering and Technology Research Center of Sichuan Province, Animtech Bioengineering Co. Ltd., Chengdu 610066, China; ^3^Livestock and Poultry Biological Products Key Laboratory of Sichuan Province, Sichuan Animal Science Academy, Chengdu, China; ^4^Animal Breeding and Genetics Key Laboratory of Sichuan Province, Sichuan Animal Science Academy, Chengdu, China

## Abstract

Porcine transmissible gastroenteritis virus is the major pathogen that causes fatal diarrhea in newborn piglets. In this study, a TGEV strain was isolated from the small intestine of diarrhea piglets in Sichuan Province, China, and designated SC2021. The complete genomic sequence of TGEV SC2021 was 28561 bp, revealing a new natural deletion TGEV strain. Based on phylogenetic analyses, TGEV SC2021 belonged to the Miller cluster and was closely related to CN strains. The newborn piglets orally challenged with TGEV SC2021 showed typical watery diarrhea. In addition, macro and micropathological changes in the lungs and intestines were observed. In conclusion, we isolated a new natural deletion virus strain and confirmed that the virus strain has high pathogenicity in newborn piglets. Moreover, macroscopic and microscopic lesions were observed in the lungs and intestines of all TGEV SC2021-infected piglets. In summary, we isolated a new natural deletion TGEV strain and demonstrated that the natural deletion strain showed high pathogenicity in newborn piglets. These data enrich the diversity of TGEV strains and help us to understand the genetic evolution and molecular pathogenesis of TGEV.

## 1. Introduction

Porcine transmissible gastroenteritis virus (TGEV) is an enveloped, single-stranded, positive-sense RNA virus that belongs to the genus *Coronavirus*, family *Coronaviridae*, and order *Nidovirales*. All pigs are susceptible to TGEV infection, with piglets under two weeks of age being particularly at risk [1]. The infected piglets mainly exhibited vomiting, diarrhea, dehydration, and mortality, while the fattened pigs showed mild diarrhea and slow growth.

The TGEV genome is approximately 28.5 kb in length and contains nine open reading frames, including ORF1a/1b, ORF2, ORF3a/3b, and ORF4-7 [2]. TGEV strains can be classified into traditional and variant strains based on whole genome analysis. The variant TGEV strains, which are characterized by gene deletions in ORF1a, ORF3a, M, and some noncoding regions, have been prevalent in the US. The traditional TGEV group can be further divided into the Miller subgroup and the Purdue subgroup [3]. Several strains belonging to the Miller and Purdue subgroup have been isolated and identified in China [4–7]. In addition, a recombinant strain between the Miller and Purdue strain has been reported in China [3, 8].

A previous study demonstrated that compared to the Purdue cluster, the Miller cluster and the variant TGEV ORF3a gene region had 29-nucleotide (nt) gene deletions [9]. More recently, Zhang reported that an 81 bp successive nucleotide deletion strain in the ORF3a gene region was identified in China, which prevented the transcription and translation of nonstructural protein 3a, but no relevant strain was isolated [10]. Isabel Sola demonstrated that deletion of the ORF3 gene in TGEV has little effect on its virulence and replication [11]. Porcine respiratory coronavirus (PRCV), a respiratory variant of TGEV, differs from TGEV by approximately 600 nucleotides of deletion in the S gene and a partial deletion in the ORF3 region, which may affect the tissue tropism and clinical disease of PRCV [12, 13]. Although the relationship between PRCV and TGEV is complex, it still reminds us that variations in the S gene and ORF3 have the potential to influence the clinical symptoms of the disease.

It is still unclear whether variation or deletion in ORF3 can affect the clinical symptoms of TGEV. In this study, we isolated and identified TGEV SC2021, followed by analyzing its genome characteristics and pathogenicity in piglets. Our genome analysis revealed that TGEV SC2021 was a natural gene deletion virus, with an 81 nt deletion including the start codon region of ORF3a in comparison with Miller subgroup strains. Compared to the newborn piglets infected with the SC-Y strain, which belonged to the Purdue subgroup, those infected with TGEV SC2021 exhibited more severe diarrhea and faster mortality, indicating that TGEV SC2021 is a highly pathogenic strain. These findings highlight that a new variant of TGEV strain is emerging which may exhibit different clinical symptoms.

## 2. Materials and Methods

### 2.1. Sample and Virus Isolation

In 2021, porcine intestines with diarrhea were collected from a swine farm in Sichuan Province, China. The small intestine sample was tested positive for transmissible gastroenteritis virus by RT-PCR and subsequently homogenized and mixed with Dulbecco's Modified Eagle Medium (DMEM). The mixture was then filtered through a 0.22 *μ*m syringe filter (Millipore, Billerica, MA, USA) and inoculated onto a monolayer of PK-15 cells. After adsorption for 2 h, the cells were incubated with fresh DMEM supplemented with 10% fetal bovine serum at 37°C in a 5% CO_2_ incubator. The inoculated cells were blindly passaged for three generations. After the discovery of the cytopathic effect (CPE), the virus was purified using the CPE plaque method. Finally, the virus titers were determined using the Reed–Muench method.

### 2.2. Identification by Reverse Transcription PCR

Total RNAs were extracted from the TGEV cell culture medium of the 5th, 10th, and 15th generations using RNAiso Plus, and reverse transcription was performed following the manufacturer's protocols. PCR was conducted with the specific primers for TGEV, rotavirus (RV), porcine deltacoronavirus (PDCoV), and porcine epidemic diarrhea virus (PEDV) (Table 1). Also, the PCR products were analyzed using 1% agarose gel electrophoresis (Sangon Biotech, Shanghai, China).

### 2.3. RNA Extraction and Complete Gene Sequencing

Total RNAs were extracted from the TGEV cell culture supernatant using RNAiso Plus, and reverse transcription was carried out according to the manufacturer's protocol. To analyze the TGEV complete gene, 22 pairs of primers targeting the complete gene of TGEV were designed and synthesized (Table 2). PCR was conducted, and the PCR products were sequenced by Sangon Biotech (Sangon Biotech, Shanghai, China). The sequences were assembled using DNAMAN (version 6.0.3.93), and the obtained sequences were submitted to the GenBank database.

### 2.4. One-Step Growth Kinetics

The replication kinetics of the TGEV SC2021 strain and the TGEV SC-Y strain were determined in PK-15 cells using the Reed–Muench method, as previously described [14].

### 2.5. Sequence Analysis

The complete genes of TGEV SC2021 were aligned with 24 TGEV reference strains from GenBank using the multiple alignments in MEGA 7.0 software. Phylogenetic trees were then constructed using the maximum likelihood method with 1000 bootstraps.

### 2.6. Electron Microscopy Observation

The PK-15 cells were infected with TGEV SC2021 and harvested at 48 hours postinfection. The supernatant was centrifuged at 5,000 g for 30 min, followed by another centrifugation at 100,000 g for 3 hours in a Beckman Optima MAX-XP and resuspended with 1 mL PBS. Then, negative staining with 2% phosphotungstic acid was performed, and the samples were imaged using a JEM-1400-FLASH transmission electron microscope.

### 2.7. Pathogenicity of TGEV SC2021 in Newborn Piglets

Fifteen one-day-old newborn piglets without infection or immunization of PEDV, TGEV, PDCoV, and PRCV were randomly assigned into three groups, each consisting of five piglets (SC2021 group, SC-Y group, and mock group). All piglets were fed with liquid milk and had free access to water. Piglets in the SC2021 group and SC-Y group were orally infected with 5 mL of 2 *∗* 10^6^TCID_50_/mL TGEV SC2021 or 5 mL of 2 *∗* 10^6^TCID_50_/mL TGEV SC-Y, respectively, while piglets in the mock group were orally treated with DMEM. All piglets were monitored daily for clinical signs of disease after the challenge. When the piglets in the challenged group appeared severe diarrhea and became moribund, they were necropsied to observe the organs' pathological changes.

### 2.8. Quantitative Real-Time PCR Assay

The tissue samples from all the piglets were collected and homogenized. Rectal swabs, which were collected every 12 hours, were suspended in 1 mL phosphate buffer saline. Total RNA was extracted using RNAiso Plus, following the manufacturer's protocol. RNA concentration and purity were measured using the A260 value and the ratio of A260/280, respectively, with a ScanDrop. Reverse transcription reactions were performed using the PrimeScript RT Kit, as previously described [15]. The viral loads in tissue samples from the challenged animals were determined with a qRT-PCR assay using the specific TGEV primers (Table 1). The gene copy number for each sample was expressed as log 10 copies per gram of tissue sample and per milliliter of rectal swabs.

### 2.9. Histopathologic Examination and Immunohistochemistry

The tissues were collected and immediately fixed with 4% paraformaldehyde solution. Then, the tissue samples were dehydrated, cleared, and embedded in paraffin. The sections were prepared and stained with hematoxylin and eosin (H and E). The lung, duodenum, jejunum, and ileum of mice fixed with 4% paraformaldehyde were sent to the Wuhan Servicebio for immunohistochemical analysis to observe the proliferation of TGEV in the intestine and lung.

The primary antibody was the mouse polyclonal antibody against the TGEV S protein, and the secondary antibody was goat anti-mouse IgG (Servicebio).

## 3. Results

### 3.1. Isolation and Identification

**Figure 1:**
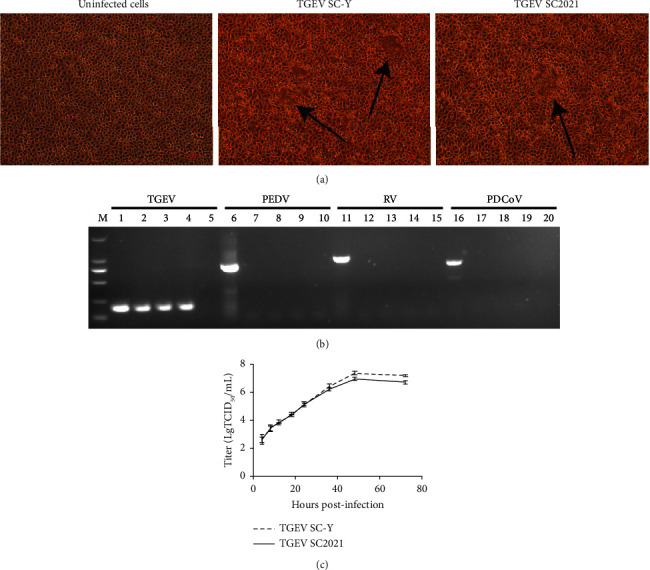
Isolation and identification of TGEV SC2021. (a) The microscopic image at 36 hours post-infection. Uninfected cells were the PK-15 cells which treated with DMEM; TGEV SC-Y was the PK-15 cells infected with TGEV SC-Y strain; TGEV SC2021 was the PK-15 cells infected with TGEV SC2021 strain. The black arrow points the CPE. (b) PCR identification results of the isolation supernatant. Lane (M) DNA Marker; lane 1: TGEV positive control; lane 2–4: 5th, 10th, 15th generations of the isolation supernatant; lane 5: TGEV negative control; lane 6: PEDV positive control; lane 7–9: 5th, 10th, 15th generations of the isolation supernatant; lane 10: PEDV negative control; lane 11: RV positive control; lane 12–14: 5th, 10th, 15th generations of the isolation supernatant; lane 15: RV negative control; lane 16: PDCoV positive control; lane 17–19: 5th, 10th, 15th generations of the isolation supernatant; lane 20: PDCoV negative control. (c) Growth kinetics of TGEV SC2021 and SC-Y with different time in PK-15 cells.

The TGEV-positive samples were inoculated with PK-15 cells, and the cells were cultured blindly for three generations. After purification by the plaque method for three times, visible CPE was observed 36 hours postinfection, including cell lysis, wire drawing, and vacuoles (Figure 1(a)). The isolation supernatant in the 5th, 10th, and 15th generations was positive for TGEV (Figure 1(b)), but negative for other porcine enteric coronaviruses. The growth features of TGEV SC2021 were analogous to those of TGEV SC-Y in PK-15 cells (Figure 1(c)). The peak titer of TGEV SC2021, which was lower than that of TGEV SC-Y, was 10^7.0^ TCID_50_/mL.

**Figure 2:**
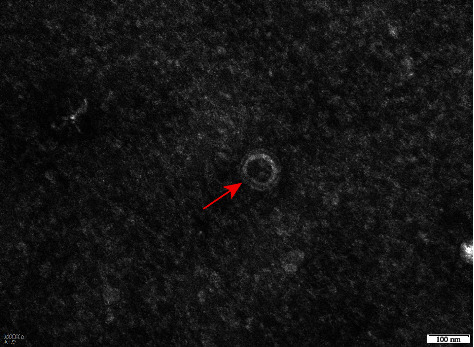
Transmission electron microscopy images of TGEV SC2021. The red arrow points the virion. Scale bars = 100 nm.

To characterize the morphology and size of the virus particles, TGEV SC2021 was imaged using transmission electron microscopy (TEM). As shown in Figure 2, typical crown-shaped particles with spiky surface projections, as in other Coronaviruses, were observed and the size of the viral particles was 100 nm in diameter, following the surface projection characteristic of Coronaviruses.

### 3.2. Complete Genome Analysis

**Figure 3:**
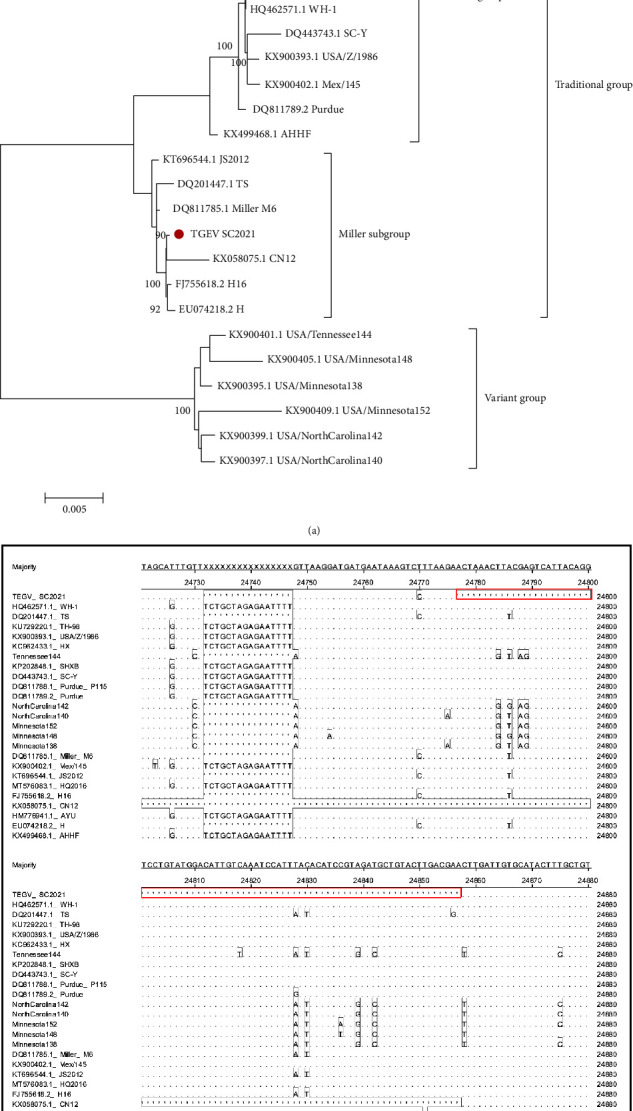
The sequence alignment and phylogenetic analysis of TGEV SC2021 complete genome. (a) Phylogenetic analysis based on TGEV SC2021 complete genome. The phylogenetic trees of TGEV SC2021 complete genome with 24 reference strains were constructed, and the red point represented TGEV SC2021. (b) Nucleotide alignment of TGEV SC2021. Alignments of the complete genome sequence with 24 reference strains were analyzed, and the 81 successive nucleotide deletions of TGEV SC2021 were marked by red solid box.

To further understand the genetic evolutionary relationship between the isolated strains and other strains, we sequenced the whole genome of TGEV SC2021 and constructed a phylogenetic tree. The results showed that SC2021 belonged to the Miller clusters located in the traditional TGEV group and was closely related to the TGEV CN12 strain isolated from Guangdong, China (Figure 3(a)). Many studies have reported that Coronavirus appears gene recombination and deletion. Therefore, we conducted a gene recombination and deletion insertion analysis on TGEV SC2021. No gene recombination was observed, but there were 81 nt deletions occurred in the ORF3a of TGEV SC2021, including the start codon region, in comparison with Miller subgroup strains. This resulted in the loss of nonstructural protein 3a (Figure 3(b)).

### 3.3. Clinical Signs Assessment

**Figure 4:**
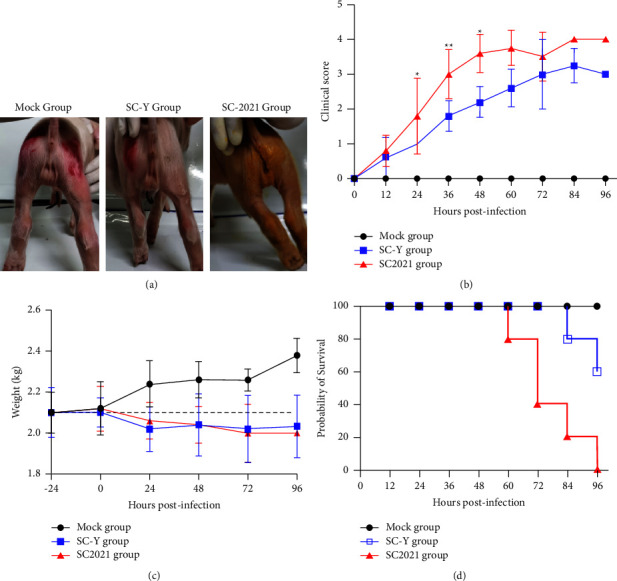
Clinical signs of newborn piglets. (a) Clinical symptoms. The defecation of piglets was observed at 24 hours post-infection. (b) Clinical score. After infected with TGEV, the fecal scores of newborn piglets were categorized into 0, normal; 1, soft; 2, very soft and tends to be liquid; 3, liquid with some solid content; 4, watery diarrhea with no solid content. (c) Weight changes of piglets in each group after challenge. Black dotted line represents initial average weight of all piglets. (d) Survival curves for newborn piglets after challenged with TGEV. The survival percentages were presented as a Kaplan-Meier plot (*n* = 5 per group).

In order to assess the pathogenicity of TGEV SC2021 in piglets, the newborn piglets were infected with isolated TGEV SC2021, and the traditional strain SC-Y was preserved in the laboratory. At 24 hours postinfection, some newborn piglets in the TGEV SC2021 group appeared semiliquid diarrhea and many fecal residues were observed in the buttocks, whereas the newborn piglets in the TGEV SC-Y group did not exhibit semiliquid diarrhea and had no fecal residues on their buttocks (Figure 4(a)). Compared to the SC-Y group, the piglets in the SC-2021 group presented with earlier and more severe clinical symptoms of diarrhea (Figure 4(b)). All piglets in the infected group experienced weight loss at 24 hours postinfection in comparison with the initial average weight, whereas the piglets in the mock group exhibited weight gain (Figure 4(c)). The newborn piglets in the TGEV SC2021 group started to die at 60 hours postinfection and all piglets in the SC2021 group died within 96 hours. On the other hand, the newborn piglets in the TGEV SC-Y group started to die after 84 hours postinfection. No deaths or clinical symptoms were observed in the mock group (Figure 4(d)).

### 3.4. Viral Shedding and Histopathological Examination of Infected Piglets

**Figure 5:**
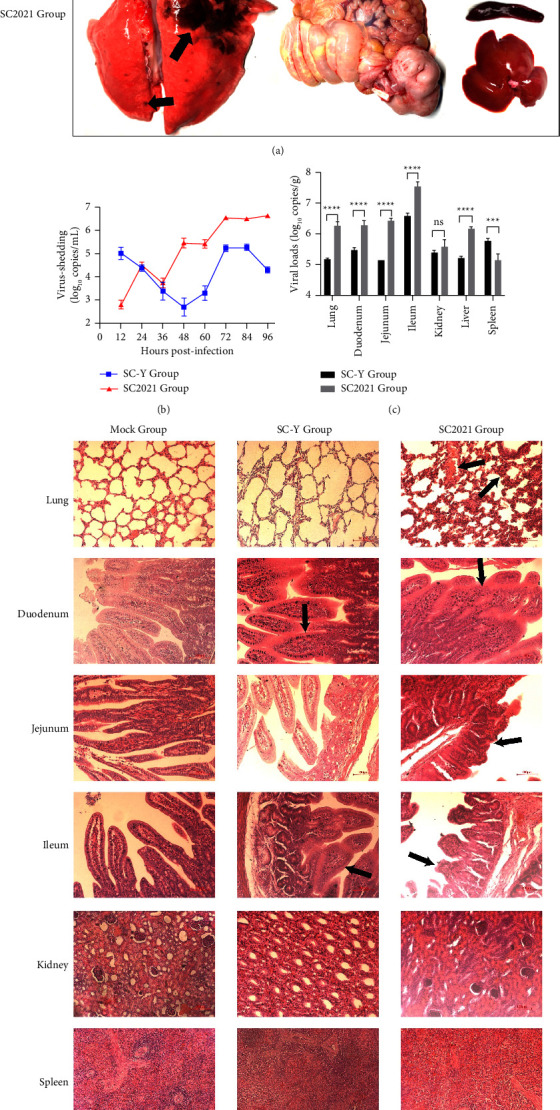
The pathogenicity of TGEV on the newborn piglets. (a) The images of gross lesions. The gross lesions of lung, intestine, kidney, liver and spleen were observed at 96 hours post-infection. The black arrow points the gross lesions. (b) The viral-shedding of piglets after infected with TGEV. The viral-shedding were represented as log 10 copies per milliliter. (c) The viral loads of intestine, lung, kidney, liver and spleen. The viral loads were represented as log 10 copies per gram. Data were presented as mean ± SD (*n* = 5). ^*∗∗∗*^*P* < 0.001, ^*∗∗∗∗*^*P* < 0.0001. (d) Histopathological analysis of luns, livers, spleen, kidney and intestine. The black arrow points thickening of the alveolar septa and fibrous exudation in the lung and villous atrophy and fusion in the intestine (hematoxylin and eosin staining, 200x magnification). (e) Analysis of lungs and intestine. The black arrow points TGEV (lung, 200x magnification; jejunum, 400x magnification).

All piglets were euthanized and organs were imaged. A significant pulmonary consolidation was observed in all piglets of the SC2021 group, in contrast to the mock and SC-Y groups. The intestines of all infected piglets were distended and filled with yellow fluid, especially in the SC2021 group. No gross lesions were observed in the organs of the mock group (Figure 5(a)). Fecal viral shedding was detected by quantitative real-time PCR (qRT-PCR). The fecal viral shedding in the SC2021 group increased over time, reaching its peak at 96 hours. In contrast, the fecal viral shedding of SC-Y-infected piglets initially decreased and reached the lowest point at 48 hours, followed by an increase and a peak at 84 hours. The peak of fecal viral shedding in the SC2021 group was higher than that of the SC-Y group (Figure 5(b)). The viral loads in the small intestine, including duodenum, jejunum, and ileum, were significantly higher in the SC2021 group than in the SC-Y group. Similarly, the viral loads of the lung and liver in the SC2021 group exceeded those in the SC-Y group. However, the viral loads in the spleen of the SC2021 group were lower than those in the spleen of the SC-Y group. There was no significant difference in the viral load of the kidney between the SC2021 group and the SC-Y group (Figure 5(c)).

Histopathological examination revealed thickening of the alveolar septa, inflammatory cell infiltration, and fibrous exudation in the lungs of the SC2021 group, while no visible histopathological damage was observed in the lungs of the SC-Y group or mock group. Compared to the mock group, necrosis and shedding of intestinal cells and villous atrophy and fusion were found in the different segments of the intestine in both the SC2021 and SC-Y groups. More severe villous atrophy was observed in the jejunum and ileum of the SC2021 group, compared to the SC-Y group. No significant histopathological damage was observed in the liver, spleen, and kidney in either the SC-Y group or SC2021 group, compared to the mock group (Figure 5(d)). In addition, immunohistochemical analysis was conducted on the lungs and intestines, and TGEV was detected in both the intestine and lung (Figure 5(e)). In conclusion, these results indicate that TGEV SC2021 may cause intestinal and lung lesions in newborn piglets.

## 4. Discussion

TGEV is the major cause of severe villous atrophy and malabsorptive diarrhea. Since 1946, outbreaks of TGEV have been reported in many countries, resulting in huge economic losses [16]. However, the situation has changed in recent years. Although a sporadic case of TGEV isolation and identification have been reported, there have been no large-scale outbreaks in China in the past 20 years [17]. Recently, many newly characterized strains of TGEV have been identified, such as recombinant strains between the Purdue and Miller clusters, as well as strains with a 6 nt deletion of the S gene strains [3, 5, 8, 18]. In our study, we isolated and identified a TGEV strain named TGEV SC2021, which has 81 successive nucleotide deletions compared with other Miller subgroup strains. Based on the alignment of the TGEV SC2021 genome sequence with 24 reference strains, the 81 successive nucleotide deletions, including a 30 bp gene deletion in the noncoding region and a 51 bp gene deletion in the ORF3a region, resulted in the loss of 3a proteins in TGEV SC2021. To our knowledge, our study is the first isolation report of an 81 successive nucleotide deletion in TGEV strains. Similarly, Zhang's study reported 81 successive nucleotide deletions in four TGEV strains, but no isolates were obtained [10]. Additionally, there was no difference in the ORF3 region between the strains isolated by our lab and the CH-SH and LNCT strains reported by Zhang's study. In summary, our findings informed us that an emerging cluster of TGEV with 81 successive nucleotide deletions is prevalent in China.

The piglets in the TGEV infection groups started to appear diarrhea and vomiting after 24 hours post infection, and loss of appetite was discovered in the TGEV SC2021 group, which is consistent with the previous study [18]. Previous studies have suggested that vomiting induced by TGEV may be caused by the neural pathway, rather than stomach damage [19]. The tissue tropisms of difference TGEV strains were different, particularly in the lungs [8, 20]. In our study, it was found that TGEV SC2021 infection in piglets caused severe lung injury, while the TGEV SC-Y strain did not induce lung damage in infected piglets. Stoian had proved that PAMs and lung fibroblasts could be infected by TGEV [21]. Although TGEV SC-Y also replicates in the upper respiratory tract and can be detected in the lung, it did not cause significant lung damage, which is consistent with previous studies [22, 23]. Interestingly, we observed severe pulmonary consolidation in TGEV-infected piglets for the first time. Pulmonary consolidation had only reported in PRCV, a respiratory variant of TGEV, infected piglets [12]. To investigate the relationship between TGEV and PRCV, we compared the full genome sequences of the TGEV SC2021 and PRCV and found that the homology was between 96.1% and 97.9% (Figure S1A). Phylogenetic analysis also revealed that TGEV SC2021 and PRCV were located in different branches (Figure S1B). Furthermore, based on the analysis of the amino acid sequences of S protein, we observed that the 220/223 amino acid-deletion observed in PRCV in comparison of TGEV SC2021 (Figure S1C), indicating that there were significant differences between the TGEV SC2021 strain and the PRCV strain. A missing start codon was identified in the ORF3a region of TGEV SC2021 and PRCV strains, resulting in the absence of the 3a protein (Figure S1D). Therefore, we speculate that the absence of ORF3a protein in the TGEV SC2021 strain may be the cause of lung injury.

The results of the viral loads in the SC2021 group revealed that the viral loads in the lungs were similar to those in the duodenum and jejunum, indicating that the lung tropism of TGEV SC2021 is comparable to its intestinal tropism. Therefore, it is possible that the observed pulmonary consolidation in the TGEV SC2021 group is due to its lung tropism. However, no significant respiratory symptoms were observed in the TGEV SC2021 infected piglets. We speculated that the respiratory symptoms of TGEV SC2021-infected piglets may be subclinical, similar to most nonacute PRCV infections [24]. In previous studies, a 6 nt deletion at positions 1123–1128 in the SC-Y strain S gene was found, which was considered to contribute to the attenuation of the virus. However, this deletion was not observed in our TGEV SC2021 strain [5]. This could be the main reason why the clinical symptoms of piglets infected with the TGEV SC-Y strain were milder compared to those infected with the TGEV SC2021 strain.

TGEV, porcine epidemic diarrhea virus (PEDV), and PRCV belong to the alpha-coronavirus (CoVs). Co-infection is common among the three alpha-coronavirus. PRCV originated from a natural gene deletion of TGEV, which included partial S genes, ORF3a, and ORF3b. Also, the antibody of PRCV could cross-protect against TGEV, which may be the reason for the gradual disappearance of TGEV [25–27]. Previous studies have demonstrated that hyperimmune TGEV Miller rather than TGEV Purdue antisera cross-reacted with PEDV strains [28]. Also, the antibody of PEDV N protein also could cross-react with TGEV. The quick spread and worldwide prevalence of PEDV and PRCV may be the main reasons for the gradual decrease of the TGEV epidemic situation. However, the recombination events between PEDV and TGEV have been discovered [25], indicating that the recombination of CoVs is occurring silently. More attention should be paid to the evolution and recombination of CoVs and prevention of a TGEV comeback. Our study suggests that a new gene deletion TGEV strain with high pathogenicity and significant lung damage is emerging. Although previous studies guessed that the ORF3a deletion caused changes in TGEV tissue tropism [10], this has been observed in the TGEV SC2021 infected piglets. The relationship between the 81 successive nucleotide deletions and tropism still needs to be proved by constructing the 81 nucleotide complemented strains. Furthermore, studies are being conducted in our laboratory to explore the effects of the successive 81 nucleotides on TGEV tropism and virulence.

## Figures and Tables

**Table 1:** Primers for reverse transcription PCR identification and viral loads detection.

Primer name	Sequence	Size/position (bp)
TGEV-F	CATAAGCAGACTGACAACAAC	174
TGEV-R	CTCACCTTTCTTTACTCCAGA	

PEDV-F	ACATTTAAACCACCAGCACTC	768
PEDV-R	TCAACGATGCCAAGCAATTGT	

PDCoV-F	CTTATCTCACTAGTTAAAAAC	923
PDCoV-R	TAGGAAGCATGTAAGTTAAAA	

RV-F	TGGATGGAGCGAACTATCTAG	1138
RV-R	AATGTAGCTGAAAAACAATTA	

**Table 2:** Primers for TGEV complete genome sequence.

Primer name	Sequence	Size/position
TGEV1-F	CGTCCTGTGATCTCCCTCGCC	1272 bp/1–1272
TGEV1-R	TCACCAGCATATTGAAGAACA	

TGEV2-F	TCTGGCAAAGTTAAGGGTGTC	1243 bp/1151–2723
TGEV2-R	TCTTACATGAATCTTTCTTTA	

TGEV3-F	TTTTGGGGAGTGTTCAACTTT	1535 bp/2553–4087
TGEV3-R	TTGAGTTGTGCCAGTGTAAAC	

TGEV4-F	TGTGGTGAAAAAGAAATTGTT	1440 bp/3860–5299
TGEV4-R	CATTAATTCAGCATCACCTGG	

TGEV5-F	TTGTCTTGCATTACAGAGACT	1494 bp/5143–6636
TGEV5-R	CCAGTACCATTAGCATGAACA	

TGEV6-F	TAGGACTGCAAGGCAGACACG	1377 bp/6550–7926
TGEV6-R	CACATATTAGTAACACCAAAA	

TGEV7-F	ATGTCCAATTGTTGTAGGAAC	1397 bp/7779–9188
TGEV7-R	TGTGAAGTCTCACACTAGACA	

TGEV8-F	CTTAATGGTTTATGGTTAGGA	1379 bp/9077–10455
TGEV8-R	ACCATCCAATCCTTTGTAGTA	

TGEV9-F	TTGTGATCTTTAACATGGTTA	1497 bp/10329–11825
TGEV9-R	CTTCAAGTTCAATAGGTATAA	

TGEV10-F	GTGGAAAAAGCTTTATGTATG	1444 bp/11721–13170
TGEV10-R	ATCATAAGCTAGTAAATCATA	

TGEV11-F	GCTTGTGTTACATCATATTAT	1343 bp/13039–14381
TGEV11-R	CCAGGTTTAAAATAAAAACCA	

TGEV12-F	GGCTTCTGCCATGATATTAGG	1365 bp/14244–15608
TGEV12-R	TTTAATACAGCATAAGCATAT	

TGEV13-F	GTCAAGGAATCTCTGAAAATT	1508 bp/15520–17027
TGEV13-R	CTCATATATGTCGTTGCATAA	

TGEV14-F	ATGTATGAGAATCTTGATTTC	1451 bp/16888–18338
TGEV14-R	CATTTTGTTATGCAAACATTT	

TGEV15-F	GCTTTCCACACACCAGCTTAT	1420 bp/18193–19612
TGEV15-R	CTTGTGCACCATAATTGTAGA	

TGEV16-F	CATGGAGATGGATGTTGTGGT	1359 bp/19454–20812
TGEV16-R	CAAATGCATATAATAGCACCT	

TGEV17-F	CGGTTAAACGTAGTCGTTAAT	1540 bp/20714–22257
TGEV17-R	CAAATGAGAAAGGACAAGTAC	

TGEV18-F	TCTTGGTATGAAGCGTAGTGG	1439 bp/22022–23463
TGEV18-R	ACATAGTCATTTTGTCAGCAT	

TGEV19-F	TCTGGTTTAGGTACAGTTGAT	1373 bp/23328–24700
TGEV19-R	TGACAACAACTTCCTAAACAA	

TGEV20-F	TATGTAAAATGGCCTTGGTAT	1461 bp/24570–26156
TGEV20-R	CAAGGAGTGCTCCATCGGGGT	

TGEV21-F	AATAGCATTGCTAAATATAAT	1380 bp/26011–27395
TGEV21-R	TCAAAGCTTTGGATTCATTAT	

TGEV22-F	ACTGGACCTCATGCAGATGCC	1376 bp/27266–28642
TGEV22-R	GTATCACTATCAAAAGGAAAA	

## Data Availability

The original contributions presented in the study are included in the article/supplementary material; further inquiries can be directed to the corresponding authors.

## Supplementary Materials

Supplementary Materials S1 Figure: The gene difference between TGEV SC2021 and PRCV.
