# Supplementary material for: Characterization and Evaluation of the Pathogenicity of a Natural Gene-Deleted Transmissible Gastroenteritis Virus in China
Source: Transbound Emerg Dis. 2023 Mar 3;2023:2652850. doi: 10.1155/2023/2652850 (PMC12017154; doi:10.1155/2023/2652850)
Supplement: Supplementary Materials — S1 Figure: The gene difference between TGEV SC2021 and PRCV. [file 2652850.f1.docx]

Figure legend:


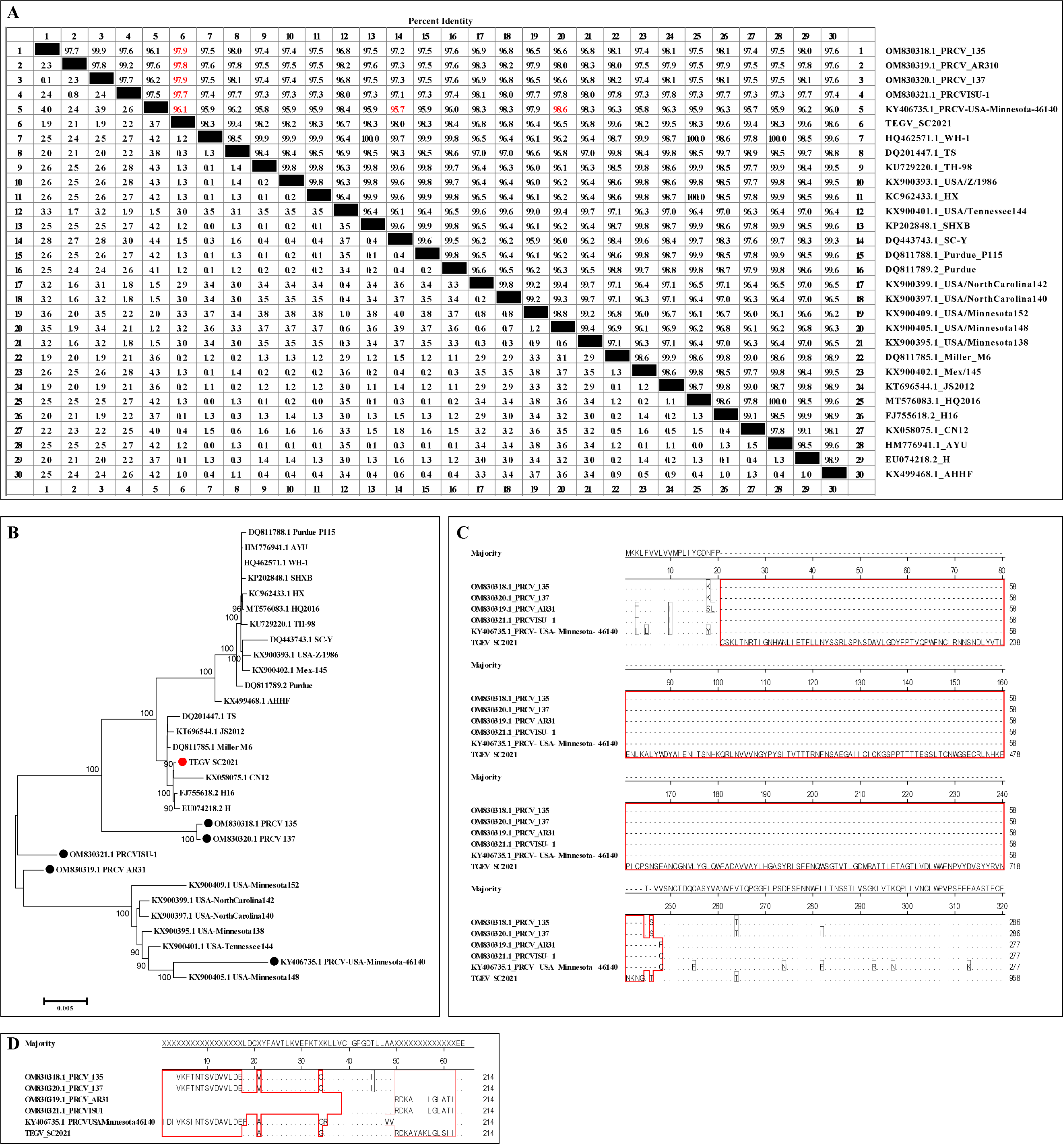


Fig. S1 The difference between TGEV SC2021 and PRCV. (A) Nucleotide alignment of TGEV and PRCV. The percent identity of TGEV SC2021 and five PRCV strains were marked by red. (B) Phylogenetic analysis. The phylogenetic trees of TGEV SC2021 complete genome and PRCV were constructed. The red point represented TGEV SC2021 and the black point represented PRCV. (C) Comparison of S gene amino acid differences between TGEV SC2021 and PRCV. The deletion/differential genes were marked by red solid box. (D) Comparison of ORF3a gene amino acid differences between TGEV SC2021 and PRCV. The deletion/differential genes were marked by red solid box.
